# Risk factors and outcomes for prolonged versus brief fever: a prospective cohort study

**DOI:** 10.1186/cc11465

**Published:** 2012-08-13

**Authors:** Philippe Seguin, Antoine Roquilly, Olivier Mimoz, Pascale Le Maguet, Karim Asehnoune, Sébastien Biederman, Elsa Carise, Yannick Malledant

**Affiliations:** 1 Département d'Anesthésie Réanimation, CHU Rennes, 2 rue Henri Le Guilloux, Rennes F-35033, France; 2 Inserm U991, 2 avenue du Professeur Léon Bernard, Rennes F-35043, France; 3 Université Rennes 1. F-35043 Rennes, France; 4 Département d'Anesthésie Réanimation, CHU Nantes, Place Alexis Ricordeau, F-44093 Nantes, France; 5 Département d'Anesthésie Réanimation, CHU Poitiers, 2 rue de la Milétrie, Poitiers F-86021, France; 6 Inserm Eri 23, 40 avenue du recteur Pineau, Poitiers F-86022, France

## Abstract

**Introduction:**

Prolonged fever occurs with infectious and noninfectious diseases but is poorly studied in intensive care units. The aims of this prospective multicenter noninterventional study were to determine the incidence and etiologies of prolonged fever in critically ill patients and to compare outcomes for prolonged fever and short-lasting fever.

**Methods:**

The study involved two periods of 2 months each, with 507 patients hospitalized ≥ 24 hours. Fever was defined by at least one episode of temperature ≥ 38.3°C, and prolonged fever, as lasting > 5 days. Backward stepwise logistic regression was performed to identify the independent factors associated with prolonged fever versus short-lasting fever.

**Results:**

Prolonged or short-lasting fever occurred in 87 (17%) and 278 (55%) patients, respectively. Infectious and noninfectious causes were found in 54 (62%) and 27 (31%) of 87 patients, respectively; in six patients (7%), prolonged fever remained unexplained. The two most common sites of infection were ventilator-associated pneumonia (*n *= 25) and intraabdominal infection (*n *= 13). Noninfectious fever (*n *= 27) was neurogenic in 19 (70%) patients and mainly associated with cerebral injury (84%). Independent risk factors for prolonged fever were cerebral injury at admission (OR = 5.03; 95% CI, 2.51 to 10.06), severe sepsis (OR = 2.79; 95% CI, 1.35 to 5.79), number of infections (OR = 2.35; 95% CI, 1.43 to 3.86), and mechanical-ventilation duration (OR = 1.05; 95% CI, 1.01 to 1.09). Older patients were less likely to develop prolonged fever. ICU mortality did not differ between the two groups.

**Conclusions:**

Prolonged fever was common, mainly due to severe infections, particularly ventilator-associated pneumonia, and mixed infectious causes were frequent, warranting systematic and careful search for multiple causes. Neurogenic fever was also especially frequent.

## Introduction

Fever is a frequent symptom in the intensive care unit (ICU), with rates ranging from 28% to 70%; its frequency depends of the types of patients evaluated, the threshold of definition of fever, and the duration of evaluation [1-4]. In a large, recent retrospective study involving more than 20,000 types of intensive care patients in the same hospital, fever ( ≥ 38.3°C) and high fever ( > 39.5°C) were reported in 44% and 8%, respectively [2].

If evaluation of new-onset fever in intensive care adult patients is well standardized [5], the problem caused by prolonged fever ( > 5 days) has not been well studied, although it has been recognized to occur in 8% to 18% of patients and is related mainly to infectious etiologies [1-3]. These findings urge us to define better the spectrum of prolonged fever in a wide population of critically ill patients. Indeed, we hypothesized that prolonged fever may have a high percentage of noninfectious causes and/or particular etiologies that could modify the care of critically ill patients.

Accordingly, the objectives of this study were to determine the incidence and etiologies of prolonged fever, to evaluate morbidity and mortality of fever, and to determine its risk factors compared with short-lasting fever in patients in the ICU.

## Materials and methods

**Figure 1:**
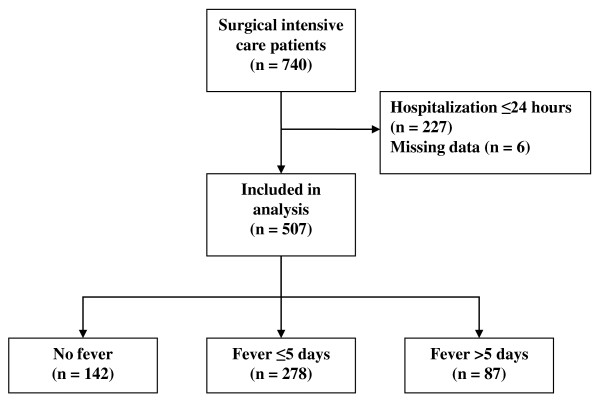
**Trial profile**.

This prospective observational study was conducted in three surgical ICUs in university hospitals in France (Nantes, Poitiers, and Rennes), and included two periods of 2 months each (May 1, 2010, to June 30, 2010, and September 1, 2010, to October 31, 2010) to avoid possible temporal etiology variations. During the two periods of study, a total of 596 patients were admitted in the ICUs, and 507 patients hospitalized ≥ 24 hours in the ICUs were analyzed (Figure 1). The local ethics committee waived informed consent because this was a noninterventional study (Comité d'éthique du CHU de Rennes, France, n°10.03).

At least every 6 hours (or more frequently if the clinical conditions required), nurses measured the patients' core temperatures at the axilla by using a digital thermometer according to the recommendations of the manufacturer (Thermofina; Z.A.C. Chamlys, Dammarie-Les-Lys, France). The temperature recorded was that provided when the acoustic signal was heard and by adding 0.5°C. When a pulmonary artery catheter was in place, the temperature recorded was obtained through this device.

At admission, the following data were recorded: age, gender, McCabe score, type of admission (medical, scheduled or nonscheduled surgery, and trauma), and presence of a cerebral injury, whatever the origin at admission. Severity was assessed by the severity acute physiology score II (SAPS II) and sequential organ failure assessment (SOFA). The presence of an infection, bacteremia, and the infected sites were also recorded.

During the ICU hospitalization, the number of infected patients, the mean number of infections per patient, the infected sites, the occurrence of bacteremia, severe sepsis or septic shock, antibiotic use and its duration, the duration of mechanical ventilation, and development of acute respiratory distress syndrome (ARDS) or acute renal failure were noted. Maximum leukocyte counts and C-reactive protein levels measured during the ICU stay were recorded. Acetaminophen (grams per day) or extracorporeal devices used (continuous renal replacement therapy or extracorporeal membrane oxygenation), known to interfere with the magnitude of the temperature, were reported. The ICU and hospital lengths of stay and ICU mortality were also recorded. The proportion of microorganisms recovered at admission and during the ICU stay were recorded and divided into those cultured from normally sterile sites (blood, mediastinum, intraabdominal, and others) and those cultured from potentially contaminated sites (urine, endotracheal tube aspirate, and others).

The search for the etiology of prolonged fever was left to the discretion of the physicians. Nevertheless, careful consideration was deemed necessary for viral infection, venous thrombosis, and evaluation of the possibility of medication-induced fever. Final diagnosis of infection was made, according to the definitions proposed by the International Sepsis Forum Consensus Conference [6] at the end of the ICU hospitalization by an experienced and independent intensivist at each center.

### Definitions

Fever was defined as at least one episode of temperature ≥ 38.3°C, and prolonged fever as that lasting at least 5 days, as previously reported [1-3,5]. Surgical patients were those who had undergone surgery in the 4 weeks preceding admission (elective surgery was a surgery scheduled > 24 hours in advance, and emergency surgery was that scheduled within 24 hours of operation). Trauma admissions were defined as ICU admissions directly related to, or occurring as a complication of, a traumatic event in the 30 days preceding admission. Neurogenic fever refers to unexplained fever in patients who had a cerebral injury, whatever the cause, and was retained after thorough investigations for infectious and noninfectious causes. Unexplained fever in other types of patients referred to patients without cerebral injury and retained after thorough investigations for infectious and non-infectious causes. Sepsis, severe sepsis, and septic shock were defined accordingly to the Bone criteria [7], and ARDS, as persistent and bilateral opacities on chest radiographs associated with a PaO_2_/FiO_2 _ratio < 200 without cardiac failure or left atrial hypertension [8]. Acute renal failure was defined as a serum creatinine concentration > 4 mg/dl (350 μ*M*), or an acute increase in serum creatinine concentration (3 × baseline value), or a urine output < 0.3 ml/kg during 12 hours or anuria during 12 hours [9].

### Statistical analysis

All statistical analyses were performed by using SAS 9.1 Statistical Software (SAS Institute, Cary, NC, USA). Values are expressed as means (standard deviation) or medians (interquartile range) and as numbers (%) as required. Patients were separated into two independent groups: prolonged fever and short-lasting fever. First, categoric variables were compared by using the χ^2 ^or Fisher Exact test. Continuous variables were compared by using the Student *t *test. For building the model in multivariate analysis, we selected variables with an external clinical judgment among those with a *P *≤ 0.20 in univariate analysis. Backward stepwise logistic regression was performed to identify the independent factors associated with prolonged fever. The calibration of the models was tested with a Hosmel-Lemeshow test. Bootstrap procedure with repeated sampling was performed to validate the model stability. Odds ratios (ORs) and 95% confidence intervals (95% CIs) were calculated. *P *< 0.05 was considered statistically significant.

## Results

Prolonged fever was observed in 87 (17%) of 507 patients. Body temperature was measured in the pulmonary artery catheter in 86 (17%) of 507 of our patients, this catheter being inserted for a mean duration of 5.4 ± 1.5 days. Table 1 lists the baseline characteristics of the patients.

**Table 1:** Characteristics at admission

		Fever		
		
	No fever (*n *= 142)	≤ 5 days (*n *= 278)	> 5 days (*n *= 87)	*P*
Age, years	58 ± 18	58 ± 18	52 ± 17	0.005
Sex, male	92 (65)	187 (67)	63 (72)	0.37
McCabe score				0.05
A	72 (51)	151 (54)	63 (72)	
B	51 (36)	97 (35)	17 (20)	
C	19 (13)	29 (10)	7 (8)	
Type of admission				0.005
Medical	34 (24)	64 (23)	15 (17)	
Scheduled surgery	44 (31)	73 (26)	10 (11)	
Unscheduled surgery	39 (27)	61 (22)	24 (28)	
Trauma	25 (18)	80 (29)	38 (44)	
Cerebral injury at admission	13 (9)	69 (25)	45 (52)	< 0.001
SAPS II	34 ± 19	42 ± 19	45 ± 16	0.16
SOFA at admission	4 ± 4	6 ± 4	8 ± 4	< 0.001
At admission				
Infection	32 (23)	72 (25)	21 (24)	0.74
Bacteremia	6 (4)	16 (6)	5 (6)	1.00
Site infected				0.98
Lungs	9 (6)	16 (6)	6 (7)	
Intraabdominal	18 (13)	30 (11)	10 (11)	
Urine	4 (3)	2 (1)	0 (0)	
Catheter	0 (0)	1 (1)	0 (0)	
Others	7 (5)	23 (8)	5 (6)	

Comparisons are given between a fever of ≤ 5 and > 5 days. Quantitative and qualitative values are expressed as mean (SD) and *n *(%), respectively. SAPS II, Simplified Acute Physiologic Score II; SOFA, Sequential Organ Failure Assessment.

Ninety-seven etiologies of prolonged fever were found in 87 patients and detailed in Table 2. Infectious and noninfectious causes were found in 54 (62%) and 27 (31%) of 87 patients, respectively, and prolonged fever remained unexplained in six (7%) patients. In 14 (16%) patients, prolonged fever was related to several causes; the main association was infective in 10 of 14 patients, and in four cases, the cause could not be distinguished between an infectious or a noninfectious cause. More than one cause of prolonged fever was significantly less frequent in patients who had shorter durations of fever (five of 278 versus 14 of 87; *P *< 0.001).

**Table 2:** Detailed etiologies for prolonged fever

Causes	
Infectious	
Ventilator-associated pneumonia	24^a^
Intraabdominal infection^b^	13
Catheter-related infection^c^	6
Postoperative mediastinitis	5
Urinary	4
Sinusitis	3
Wounds	3
Others^d^	12
Noninfectious	
Neurologic	19
Thrombosis	4
Medications	1
Others^e^	3
Unknown	6

Mixed causes	
Infectious	10
2	9
3	1
Infectious and noninfectious	4

Values are expressed as etiologies per patient. ^a^One patient experienced two episodes of ventilator-associated pneumonia; ventilator-associated pneumonia, *n *= 25. ^b^Postoperative peritonitis, *n *= 10; intraabdominal abscess, *n *= 1; pancreatitis, *n *= 1; and colitis, *n *= 1. ^c^Central venous catheter, *n *= 5, and arterial catheter, *n *= 1. ^d^Prosthetic infection, *n *= 2; cellulitis, *n *= 2; viral, *n *= 2; aspiration, *n *= 2; empyema, *n *= 1; Lyell syndrome, *n *= 1; endocarditis, *n *= 1; and pharyngeal abscess, *n *= 1. ^e^Hemophagocytic syndrome, *n *= 1; hematoma resorption, *n *= 1; and arthritis, *n *= 1.

**Figure 2:**
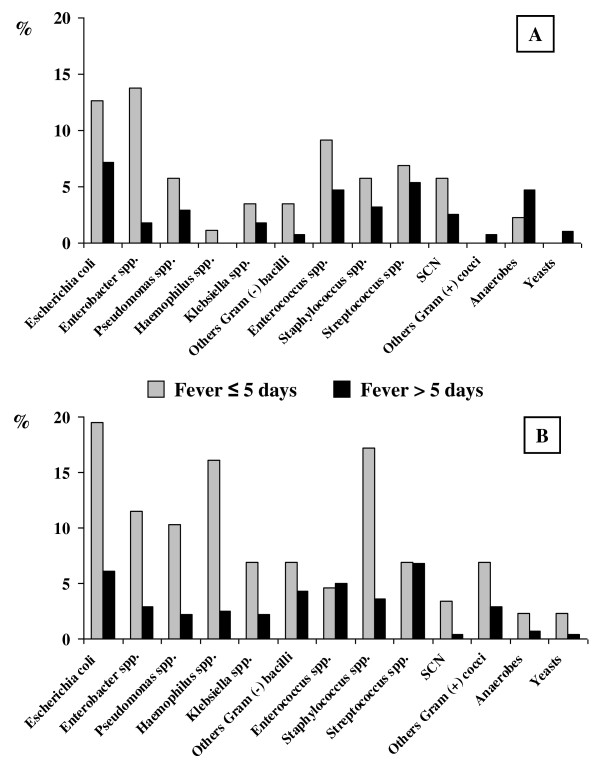
**Proportion of microorganisms recovered from normally sterile sites (A) and those cultured from potentially contaminated sites (B)**. SCN: *Staphylococcus *coagulase negative. Viruses are not represented (none recovered from sterile sites and two from potentially contaminated sites in the prolonged-fever group).

The two most common types of infection were ventilator-associated pneumonia (VAP) and intraabdominal infection. VAP was associated with one (*n *= 9) or two (*n *= 1) other causes for prolonged fever, mainly another infection (seven of 10). Intraabdominal infection was the only cause of prolonged fever in 10 of 13 patients. Catheter-related infection was the third most common infectious cause of prolonged fever, but in four of six patients, it was associated with other possible causes (intraabdominal infection, *n *= 2; VAP, *n *= 2; and venous thrombosis, *n *= 1). The proportion of microorganisms recovered at admission and during ICU stay between those cultured from normally sterile sites and those cultured from potentially contaminated sites is displayed in Figure 2.

In noninfectious causes of fever, neurogenic fevers were predominant, observed in 19 of 27 patients and mainly (16 of 19) in those who had cerebral injury at admission (Table 2). All but one case of neurogenic fever was the only cause recognized for prolonged fever. A thrombosis was found in four patients, but considered the only cause of prolonged fever in only one patient (arterial thrombosis).

Univariate analysis is presented in Tables 1 and 3. In patients who had cerebral injury at admission, the incidence of neurogenic fever did not differ between trauma and nontrauma patients, whereas infections were more frequently observed in trauma patients (eight of 30 versus eight of 15; *P *= 0.152; and 20 of 30 versus five of 15, *P *= 0.071, respectively). Finally, ICU and hospital lengths of stay were increased, whereas ICU mortality rates did not differ between the two groups (Table 4).

**Table 3:** Clinical and biologic data during ICU hospitalization

		Fever		
		
	No fever (*n *= 142)	≤ 5 days (*n *= 278)	> 5 days (*n *= 87)	*P*
No. infected patients	35 (25)	133 (49)	59 (68)	0.002
No. infections/patient	0.3 ± 0.6	0.6 ± 0.7	1.6 ± 1.0	< 0.001
Site infected				0.84
Lungs	8 (6)	99 (36)	53 (61)	
Intraabdominal	9 (6)	21 (8)	5 (6)	
Urine	0 (0)	26 (9)	15 (17)	
Catheter	0 (0)	14 (5)	11 (13)	
Others	18 (13)	43 (15)	16 (18)	
Bacteremia during ICU stay	2 (1)	32 (12)	31 (36)	0.004
Severe sepsis	21 (15)	62 (22)	49 (56)	< 0.001
Septic shock	15 (11)	54 (19)	27 (31)	0.03
Antibiotic use	31 (22)	119 (43)	75 (86)	< 0.001
Antibiotic duration, days	2 ± 4	4 ± 6	13 ± 14	< 0.001
Mechanical ventilation duration, days	2 ± 3	7 ± 9	21 ± 19	< 0.001
ARDS	6 (4)	19 (7)	20 (23)	< 0.001
Acute renal failure	29 (20)	75 (27)	23 (26)	0.9
Acetaminophen use, g/day	1.0 ± 1.4	0.7 ± 0.2	1.0 ± 1.3	0.02
Extracorporeal devices use	8 (6)	39 (14)	15 (17)	0.57
Venous thrombosis	0 (0)	13 (5)	9 (10)	0.052
Maximum leukocyte count, Giga/L	15.0 ± 7.3	18.8 ± 13.2	20.3 ± 11.7	0.38
Maximum CRP level, mg/L	121 ± 99	155 ± 114	158 ± 103	0.9

Comparisons are given between a fever of ≤ 5 and > 5 days. Quantitative and qualitative values are expressed as mean (SD) and *n *(%), respectively. ICU, intensive care unit; ARDS, acute respiratory distress syndrome; CRP, C-reactive protein.

**Table 4:** Lengths of stay and ICU mortality

		Fever		
		
	No fever (*n *= 142)	≤ 5 days (*n *= 278)	> 5 days (*n *= 87)	*P*
Lengths of stay, days				
ICU	5 ± 4	10 ± 9	27 ± 24	< 0.001
Hospital	25 ± 30	27 ± 27	53 ± 42	< 0.001
ICU mortality	18 (13)	53 (19)	13 (15)	0.38

Comparisons are given between a fever of ≤ 5 and > 5 days. Quantitative and qualitative values are expressed as mean (SD) and *n *(%), respectively. ICU, intensive care unit.

Age, SOFA, MacCabe, cerebral injury at admission, severe sepsis, septic shock, ARDS, thrombosis, number of infections during ICU hospitalization, and duration of mechanical ventilation were variables entered for the multivariate analysis. Independent risk factors for prolonged fever were cerebral injury at admission (OR = 5.03; 95% CI, 2.51 to10.06), severe sepsis (OR = 2.79; 95% CI, 1.35 to 5.79), number of infections (OR = 2.35; 95% CI, 1.43 to 3.86), and mechanical ventilation duration (OR = 1.05; 95% CI, 1.01 to 1.09). The model predicted well the risk for prolonged fever (C-index = 0.89). Age was protective (OR = 0.97; 95% CI, 0.96 to 0.99), with older patients being less susceptible to developing prolonged fever.

## Discussion

In this prospective study involving a large population of critically ill patients, 17% of patients experienced prolonged fever. Infections were the leading cause, and VAP, the main infection; the number of infections, the presence of severe sepsis, and mechanical ventilation duration were independent risk factors. Noninfectious causes were mainly neurogenic, and cerebral injury at admission was an independent risk factor. Finally, older patients were less susceptible to prolonged fever. Prolonged fever did not affect the mortality when compared with shorter-lasting fever.

Few studies have evaluated the incidence of prolonged fever in critically ill patients, and none has specifically focused on it. Notably, none described the precise etiologies and evaluated the clinical relevance. In 100 consecutive patients, prolonged fever was reported in 16%; an infective cause was recognized in all patients [1]. More recently, in a prospective study in 493 patients, prolonged fever was found in 8% and was also reported to be due to an infectious cause in 74% [3]. Finally, in a retrospective study involving more than 20,000 patients, prolonged fever was found in 18% [2]. The strength of our study lies in its multicenter and prospective design, performed during two time periods to avoid possible temporal etiology variations, and precise description of causes. Prolonged fever was present in 17%. Infection was the main cause, but less frequently than previously reported [1,3]. The first interesting and surprising finding is that the most common infective site involved was related to VAP, followed by intraabdominal sites. Nevertheless, one report indicated that times to resolution of temperature, improvement of the PaO_2_/FiO_2 _ratio, and improvement in leukocyte counts after antimicrobial therapy in patients with VAP were low; the mean time to resolution of these clinical parameters was 6 days [10]. More recently, another study reported that in patients who had VAP with and without ARDS, fever (≥ 38°C) was still present in 85% and 25% after 72 hours, respectively, despite appropriate antimicrobial therapy [11].

In our study, the duration of mechanical ventilation was an independent risk factor for prolonged fever and must be interpreted in light of the high incidence of VAP, which in turn, prolonged the need for mechanical ventilation.

The second finding that merits emphasis is the multiple causes of infective prolonged fever. Accordingly, our finding argues for systematically searching for another cause of prolonged fever, notably concomitant infection. Finally, according to the high infection rate observed in patients with prolonged fever, it not surprising that in multivariate analysis, the number of infections was an independent risk factor.

Severe sepsis was an independent risk factor for prolonged fever. Proinflammatory cytokines (endogenous pyrogens) have a central role in the genesis of fever, and high and prolonged levels of cytokines are expressed in patients with severe sepsis [12,13]. The fact that severe sepsis favors prolonged fever more than septic shock, which produces the highest cytokines levels, is surprising. Nevertheless, in about 10% of the most severely ill sepsis patients, the febrile response was blunted despite an augmented cytokine response, when compared with that in febrile patients [14].

In our study, involving a heterogeneous population of neurologic patients in the ICU, neurogenic fever was the second and the leading noninfectious cause for prolonged fever. Neurogenic fever was observed in 84% of patients who had cerebral injury, and this explains why cerebral injury at admission was found to be an independent risk factor. Fever in neurologic ICU patients is common, and its incidence ranged from 22% to 47% [15,16]. Specifically, neurogenic fever was reported in 28% and 29%, and preferentially in patients with subarachnoid hemorrhage [15,17]. Our results are in accordance with such findings and emphasize the value of neurologic injury as a cause of prolonged fever, but we did not find a difference between traumatic and nontraumatic brain injury.

Among risk factors for prolonged fever, older patients were less susceptible to developing prolonged fever. Thermoregulation mechanisms are well preserved in the elderly [18]. Nevertheless, basal temperatures are lower in older people and in face of an infectious/inflammatory process, both experimental and human data argue for a lower febrile response in these patients, which may explain our result [3,19-21].

Interestingly, mortality did not differ between patients who had prolonged fever and those who had short-lasting fever. Our results do not agree with those in two studies that found a higher mortality rate in patients who had prolonged fever [1,3]. Nevertheless, the population we studied differed because we had a higher proportion of patients with cerebral injury or trauma or both. Fever has a detrimental effect on outcomes in patients with cerebral injury. Nevertheless, prolonged fever did not seem to worsen the prognosis when compared with a shorter duration of fever. Moreover, VAP represented the main infective cause of prolonged fever in our patients, and some studies have shown no evidence of an attributable mortality to VAP in trauma patients [22,23].

Our study has some limitations. The definition of prolonged fever could be disputed. The end point of 5 days may appear too rigid, and it could be more relevant to consider prolonged fever as one that persists after well-conducted antimicrobial therapy in cases of infection or after a negative exhaustive screening search for infection. Nevertheless, three epidemiologic studies previously retained the threshold of 5 days for prolonged fever in the ICU. The definitions and method we used to determine infectious and noninfectious causes of fever may lead to over- or underdiagnoses, particularly in some circumstances in which the diagnosis is difficult and/or nonspecific definitions exist (in VAP, in particular). Conversely, all the data were reviewed at the end of each hospitalization, and according to the definitions proposed, by an experienced and independent intensivist at each center, and we believe that such an approach allows reducing the errors that could be inherent in the design of our study. The method of temperature measurements we used could be criticized; the optimal site of core temperature is provided by the pulmonary artery catheter, although this method is limited to patients requiring such a device [5]. In critically ill patients, the temperature-measurement site is controversial, and in fact, when compared with other sites in adult patients in the ICU, the axillary method remains accurate enough to be used in clinical practice [24-26]. Moreover, it has been proposed that axillary measurement with a digital thermometer has several advantages: it is easy to use, comfortable, secure, inexpensive, and durable [27-29].

Finally, this study was performed in surgical intensive care units, and our results could not be extrapolated to medical patients, who are underrepresented.

## Conclusions

Prolonged fever is common in critically ill patients and is mainly due to severe infections, particularly VAP. Mixed causes were frequently recognized, notably infective, and such an association warrants systematic and careful search for causes. Neurogenic fever was a frequent cause of prolonged fever in patients with cerebral injury and often observed as the only cause. Mortality did not differ from shorter lasting fever.

## Key messages

• Prolonged fever is common in critically ill patients.

• Ventilator-associated pneumonia is the leading cause of prolonged fever.

• More than one infectious or noninfectious cause must be carefully researched.

• In patients with cerebral injury, neurogenic fever was a frequent cause of prolonged fever and often observed as the only cause.

## Abbreviations

ARDS: Acute respiratory distress syndrome; ICU: intensive care unit; SOFA: Sequential Organ-failure Assessment; SAPS II: Severity Acute Physiology Score II; VAP: ventilator-associated pneumonia.

## Competing interests

The authors declare that they have no competing interests.

## Authors' contributions

PS made an important contribution to the conception and interpretation of the data and wrote the final manuscript version. AR, OM, and KA made an important contribution to acquisition, data analysis, and to the final manuscript version. SB and EC made an important contribution to acquisition and data analysis. PLM made a substantial contribution in the statistical design and analysis and acquisition of data. YM made an important contribution to the final manuscript version. All authors read and approved the manuscript for publication.
